# Therapeutic TG2 inhibition reverses systemic multiomic dysregulation in celiac disease

**DOI:** 10.1186/s12916-026-04892-y

**Published:** 2026-04-24

**Authors:** Valeriia Dotsenko, Hana Hien Le, Sonja Rajić, Robert Moulder, Jalmari Kettunen, M. Karoliina Hirvonen, Alex M Dickens, Tuulia Hyötyläinen, Bernhard Tewes, Timo Zimmermann, Ralf Mohrbacher, Tomi Suomi, Terho Lehtimäki, Riitta Lahesmaa, Matej Orešič, Laura L. Elo, Emma Raitoharju, Detlef Schuppan, Markku Mäki, Keijo Viiri

**Affiliations:** 1 Celiac Disease Research Center, Faculty of Medicine and Health Technology, Tampere University, Tampere University Hospital, Tampere, Finland; 2 Molecular Epidemiology, Faculty of Medicine and Health Technology, Tampere University, Tampere, Finland; 3 Turku Bioscience Centre, University of Turku and Åbo Akademi University, Turku, Finland; 4 InFLAMES Research Flagship Center, University of Turku, Turku, Finland; 5 School of Science and Technology, Örebro University, Örebro, Sweden; 6 Dr. Falk Pharma GmbH, Freiburg, Germany; 7 Department of Clinical Chemistry, Fimlab Laboratories, and Finnish Cardiovascular Research Center, Tampere, Finland; 8 Faculty of Medicine and Health Technology, Tampere University, Tampere, Finland; 9 Institute of Biomedicine, University of Turku, Turku, Finland; 10 School of Medical Sciences, Faculty of Medicine and Health, Örebro University, Örebro, Sweden; 11 Department of Life Technologies, University of Turku, Turku, Finland; 12 Tays Research Services, Wellbeing Services County of Pirkanmaa, Tampere University Hospital, Tampere, Finland; 13 Fimlab Laboratories, Tampere, Finland; 14 Institute of Translational Immunology and Celiac Center, Medical Center, Johannes-Gutenberg University, Mainz, Germany; 15 Division of Gastroenterology, Beth Israel Deaconess Medical Center, Harvard Medical School, Boston, MA USA; 16 Faculty of Biochemistry and Molecular Medicine, University of Oulu, Oulu, Finland

**Keywords:** Celiac disease, Plasma lipidomics, Biomarkers, Transglutaminase 2, DNA methylation, Proteomics, Epigenetics

## Abstract

**Background:**

Celiac disease (CeD) is an autoimmune disease triggered by dietary gluten in genetically predisposed individuals. Deamidation of gluten peptides by the CeD autoantigen and enzyme transglutaminase 2 (TG2) is central to the pathogenesis of CeD. Inhibition of TG2 with the specific inhibitor ZED1227 effectively prevents gluten-induced histological damage in CeD patients. Here we aimed to explore the systemic plasma lipidomic, proteomic and DNA methylomic changes in ZED1227-treated CeD patients undergoing a gluten challenge.

**Methods:**

Individuals with CeD on a long-term gluten-free diet (GFD) underwent a 6-week gluten challenge combined with daily 100 mg ZED1227 drug (PGCd, *n* = 28) or placebo (PGCp, *n* = 19). Samples were collected at baseline (GFD) and post-gluten challenge (PGC). Mass spectrometry-based lipidomic and proteomics profiling were applied to plasma samples matched with duodenal histology. Whole blood samples (drug, *n* = 20; placebo, *n* = 16) were subjected to DNA methylation analysis. Comparative analyses were performed between the groups, with adjustment for BMI, age, sex, and country of origin.

**Results:**

Significantly different gluten-induced plasma lipidomic changes were detected between GFD vs. PGCp and between GFD vs. PGCd, with 46 lipids differentially expressed in the placebo group and 6 in the drug group suggesting that the ZED1227 normalized gluten-induced lipidomic changes in plasma. Changes in medium-chain fatty acylcarnitines (CARs), particularly CAR 10:1 and CAR 9:0, were correlated with transient, non–clinically significant changes in renal biomarkers, with kidney function remaining within the normal range in the PGCp group. Glomerular filtration rate and plasma creatinine were restored with ZED1227. Integrated multi-omics analysis revealed a coordinated immune–epigenetic–lipid module centered on Ficolin-2, PUFA-enriched triglycerides, and a tightly co-regulated CpG cluster in the *PER3* circadian regulator gene, highlighting selective immunometabolic coupling independent of clinical stratification. Drug treatment revealed consistent patterns suggesting normalization of the proteome and DNA methylome indicating that ZED1227 attenuated the systemic responses to gluten challenge.

**Conclusions:**

These findings provide evidence that ZED1227 can significantly prevent the gluten-induced CeD-associated systemic changes in plasma/blood.

**Clinical trial:**

EudraCT 2017-002241-30.

**Supplementary Information:**

The online version contains supplementary material available at 10.1186/s12916-026-04892-y.

## Background

Celiac disease (CeD) is an immune-mediated small intestinal disorder in genetically susceptible individuals, in which dietary gluten acts as a disease-driving antigen through pathogenic T cell responses. CeD is a complex, multifactorial disease marked by a T-cell mediated immune response to gluten peptides which are resistant to complete digestive degradation due to their high proline and glutamine content [1]. Genetic susceptibility plays a key role in CeD pathogenesis, with the human leukocyte antigen (HLA) class II molecules-particular HLA-DQ2 and HLA-DQ8 heterodimers-being the primary genetic determinants. Globally, CeD affects about 1.4% of the population based on serology with biopsy-confirmed cases at 0.7%, with rising prevalence [2]. This highlights the critical need for early detection and effective dietary management of CeD [3].

Patients, both children and adults, can exhibit a spectrum of inflammatory enteropathy, clinically being asymptomatic or presenting as a severe malabsorptive syndrome, often with extraintestinal manifestations with or without gastrointestinal symptoms [4, 5]. Certain molecular mechanisms of CeD remain elusive despite progress in genetic and immunological research [6, 7]. In patients without or delayed therapy, i.e., the gluten-free diet (GFD), CeD can lead to high morbidity and even mortality, contributing to complications such as neurological disorders [8–10], osteoporosis [11, 12], kidney diseases, [13–15] autoimmune endocrine diseases, [16] especially type 1 diabetes and autoimmune thyroiditis [17], and various malignant complications [18–20]. Even with adherence to the GFD, up to 30% of patients continue to suffer from CeD-related symptoms [21], often confirmed by histological signs of inflammation and villous atrophy [22].

Although strict adherence to the GFD usually improves major intestinal and some extraintestinal manifestations of CeD, maintaining this diet is difficult due to inadvertent ingestion of traces of gluten that are present in most prepared foods, resulting in continuing symptoms [23], long-term small intestinal mucosal injury [24], adversely affecting health and quality of life [25, 26]. In light of these challenges, the past decade has seen significant research efforts directed toward developing pharmacological treatments for CeD [27, 28]. A promising advance in this field is ZED1227, an oral, first-in-class selective inhibitor of transglutaminase 2 (TG2). ZED1227 is a selective, active-site inhibitor of tissue transglutaminase (TG2) that inhibits its Ca²⁺-dependent transamidation activity, including protein crosslinking and gluten peptide deamidation, which are central to celiac disease pathogenesis.This TG2 inhibitor has shown safety and efficacy in Phase 1 clinical trials [29]. Histological outcomes from the parent clinical trial showed that ZED1227 significantly attenuated gluten-induced mucosal injury, as assessed by changes in villous height–to–crypt depth ratio and intraepithelial lymphocyte counts, compared with placebo [30, 31].

There has been a growing interest in understanding metabolic and lipidomic alterations in CeD. Recent efforts have focused on identifying metabolic alterations in the serum of potential adult [32] and pediatric [33, 34] CeD patients, including signatures that may precede the clinical manifestations. The GFD has been shown to improve metabolic [35] and lipid profiles, which are particularly affected by CeD [36]. However, there is a lack of research on the lipidomic markers in the blood of CeD patients undergoing a gluten challenge, especially when combined with effective drug treatment.

Recent studies of CeD biopsies suggest that proteomic data may aid in diagnosing morphological changes in the duodenal mucosa [37, 38]. Furthermore, a plasma proteomic study has suggested differentially expressed proteins that may serve as potential diagnostic biomarkers for CeD [39]. Importantly, only one study has suggested that blood-based DNA methylation biomarkers may be useful in detecting chronic gastrointestinal disorders such as CeD [40]. While gluten exposure triggers an immediate systemic cytokine response, most notably an increase in interleukin-2 (IL-2) [41, 42], there are currently no published studies investigating the long-term effects of gluten-induced changes in the plasma proteome and DNA methylome in conjunction with pharmacological treatment in CeD.

Using blood samples collected before and after a six-week gluten challenge in CEC3 trial of CeD patients, we performed a post hoc exploratory analysis to characterize systemic plasma lipidomics, and proteomics, and blood cell DNA methylation changes associated with gluten exposure in patients on a gluten-free diet. The cohort was designed to assess attenuation of gluten-induced duodenal mucosal injury, and the present analyses were restricted to participants receiving the highest ZED1227 dose (100 mg) or placebo. We sought to detect systemic molecular signatures during a gluten challenge and explored whether treatment with ZED1227 modulated these systemic responses compared with placebo.

## Methods

### Patient recruitment, study design, sample collection and processing

This study utilized plasma-EDTA samples from a multi-site, double-blind, randomized, placebo-controlled trial designed to determine the optimal dose and assess the efficacy and tolerability of a 6-week treatment with TG2-inhibitor ZED1227 capsules versus placebo in subjects with well-controlled CeD undergoing a gluten challenge (EU Clinical Trials Register, EudraCT Number: 2017-002241-30). Blood samples were collected following an overnight fast into EDTA-containing tubes, processed according to standardized clinical trial procedures, and plasma was separated by centrifugation and stored at − 80 °C until analysis. Sample collection, processing, and storage were performed prior to the current post hoc analyses and are consistent with established best practices for plasma-based metabolomic and lipidomic studies.

The full inclusion and exclusion criteria have been published [30]. Briefly, patients had a biopsy-proven CeD diagnosis, reported a strict GFD for at least one year, were symptom-free, showed normalized duodenal histology compared to their initial diagnostic biopsy, and tested negative for TG2 antibodies at study inclusion (GFD group, Table 1). These patients were then challenged with a cookie containing 3 g of gluten daily for 6 weeks (PGC group). Compliance of at least 80% was confirmed. Comparisons of demographic and histological characteristics of the present study cohort to original [30] is shown in Additional file 2: Table S1 and Table S2.

**Table 1:** Demographics and characteristics of patients

Variables	Lipidomic and Proteomics cohort		Methylomics cohort	
	Drug (d, *n* = 28)	Placebo (p, *n* = 19)	Drug (d, *n* = 20)	Placebo (p, *n* = 16)
Sex, no. (%)				
Male	10 (35.7%)	7 (36.8%)	7 (35.0%)	6 (37.5%)
Female	18 (64.3%)	12 (63.2%)	13 (65.0%)	10 (62.5%)
Age, year				
Mean ± SD	40.9 ± 15.0	45.3 ± 14.8	40.7 ± 15.1	46.8 ± 14.6
Range	22–65	19–62	23–64	23–62
BMI, kg/m^2^				
Mean ± SD	25.2 ± 4.1	25.1 ± 4.5	24.9 ± 4.1	25.2 ± 4.9
Range	18.7–35.4	18.7–34.5	20.4–35.4	18.7–34.5
TG2 IgA, kU/L				
Median(Q1-Q3), at the baseline	1 (1-2.25)	1 (1-1.5)	1 (1-2.5)	1 (1-1.25)
Median(Q1-Q3), post gluten challenge	1 (1–2)	1 (1–7)	1 (1–2)	1 (1-6.25)

Blood samples were collected from each participant at two defined time points: at study inclusion (baseline, referred to as Gluten free diet, GFD) and at the final visit post gluten challenges (PGC) (Additional file 2: Fig. S1). Plasma-EDTA was separated from the cell pellet and stored at -80 °C.

The study analyzed samples from two groups, both before (GFD) and after the gluten challenge (PGC): the placebo arm (p) and the 100-mg ZED1227 drug arm (d) groups. The latter, being the highest dose group, showed the most significant improvement compared to the placebo and was thus selected for the current study. A total of 47 patients (drug group, *n* = 28; placebo group, *n* = 19, with a total of 94 GFD baseline and PGC samples) were included from the original 68 patients who had adequate biopsy samples at both time points [30] and who provided separate written informed consent for these exploratory (optional) studies.

### Concomitant medications

Information on concomitant medication use was collected during the clinical trial and reviewed for the current analysis. Medications with known or potential relevance to lipid metabolism were summarized by treatment group, including lipid-modifying agents (like statins), thyroid hormone therapy, hormonal contraception, proton pump inhibitors, and antidiabetic medications (Table 2). Concomitant medications were not restricted by the study protocol, and their use reflects standard clinical care in the study population. Detailed dietary intake data were not collected as part of the original clinical trial.

**Table 2:** Concomitant medications with potential relevance to lipid metabolism

Concomitant medications	Drug group (*n* = 28)	Placebo group (*n* = 19)
**Lipid modifying agents**	3 (10.7%)	0 (0.0%)
Atorvastatin	1 (3.6%)	0 (0.0%)
Simvastatin	1 (3.6%)	0 (0.0%)
Omega 3 fish oil	1 (3.6%)	0 (0.0%)
**Thyroid therapy**	4 (14.3%)	4 (21.1%)
**Hormonal contraception**	7 (25.0%)	3 (15.8%)
**Proton pump inhibitors**	1 (3.6%)	2 (10.5%)
**Drugs used in diabetes**	0 (0.0%)	1 (5.3%)

### Lipidomics

A total of 94 plasma samples were randomized and subjected to lipid extraction using a modified version of the Folch procedure [43]. Promptly after extraction, 10 µL of 0.9% NaCl and 120 µL of CHCl_3_:MeOH (2:1, v/v) containing 2.5 µg/mL internal standard solution (for quality control and normalization purposes) were added to 10 µL of each plasma sample. A detailed protocol of plasma lipid profiling and data preprocessing is given in the Additional File 1: Supplementary Methods.

### DNA methylation

Genomics DNA was extracted from whole blood samples available from 20 drug group and 16 placebo group patients with Chemagic 360 robot with CMG-1091 kit (Revvity, Waltham, MA, USA) in combination with chemagic™ DNA Blood 400 kit H96 (Cat. No. CMG-1091), following the manufacture’s protocol (VD190913.che). DNA methylation profiling was performed using the Illumina Infinium MethylationEPIC version 2 BeadChip at Helmholtz Zentrum, Munich, Germany. Samples were applied to the arrays in a randomized order. Aliquots of 1 µg of DNA were subjected to bisulfite conversion, and 4 µl of bisulfite-converted DNA underwent whole-genome application, enzymatic fragmentation, and hybridization onto the MethylationEPIC BeadChip (EPICv2). The arrays were scanned using the iScan system (Illumina, San Diego, CA, USA).

### Proteomics

The samples were randomized for their preparation and analysis as follows. Plasma protein digests were prepared in a 96-well plate format. Briefly, aliquots (4 µl) were diluted and denatured with urea, reduced with dithiothreitol, alkylated with iodoacetamide, and digested with trypsin (at 1:30 ratio). The digests were acidified, then desalted and concentrated using C18 solid-phase extraction (SepPak C18, Waters) as described previously [44]. Samples were analysed with a Q Exactive HF Orbitrap mass spectrometer (Thermo Scientific) coupled to an Evosep One liquid chromatograph (Evosep) using the 30 samples per day method. The samples were divided into three randomized batches, with a QC sampke, composed of a pool of plasma digests, analysed periodically between the batches. Further method details can be found in the Additional file 1: Supplementary Methods.

### Statistical analysis

The statistical analysis was performed using R (Version 4.3.0 (2023-04-21)), R Foundation for Statistical Computing (Vienna, Austria).

#### Lipidomics analysis

1007 molecular features were detected. For statistical analysis and biological interpretation, only structurally annotated lipids (*n* = 308) were included, while unidentified features were excluded. Zero intensity values were considered as non-detected and treated as missing. Missing values were imputed with the minimal detected expression value. Lipid detection completeness was high, with a median of 308 lipids detected per sample and min-max range 305–308 (Additional file2: Table S3). No additional filtering based on missingness was applied, and all 308 identified lipids were retained for downstream analyses. The data underwent normalization using the autoscaling method, which involved mean centering and subsequent scaling by standard deviation. Significantly expressed lipids were identified using the Limma package. The model matrix included the sample group, patient BMI, age, and country as covariates. To account for patient-to-patient variability, patient ID was treated as a random effect. A p-value below 0.05, without adjustment for multiple testing, was considered statistically significant. Lipid set Enrichment analysis was conducted utilizing the fgsea package [45] (version 1.28.0) with logFC serving as the ranking statistic for lipids, and structural classification was used for grouping information.

#### DNA methylation analysis

The DNA methylation data was pre-processed using the default meffil pipeline, with a p-value threshold of 0.05. Probes that failed quality control (QC) were removed from further analysis. Principal components were derived from control probes and estimated cell type proportions were calculated for drug and placebo groups separately using the meffil package. An epigenome-wide association study (EWAS) was performed to assess differences between baseline and post-exposure conditions, correcting the model with age, sex, the first 20 principal components and estimated cell type proportions. Analyses were performed separately for the drug and placebo groups. Comparisons of cell type proportions between the placebo and drug groups were performed at both baseline and post-exposure using Mann-Whitney U test. In addition, Spearman correlation analysis was conducted to assess CpG sites exhibiting a change in beta values greater than 2.5% in both the drug and placebo conditions across different timepoints, as well as between treatment groups at the challenge phase.

#### Proteomics analysis

Using Spectronaut software (version 19), the data were searched with the direct DIA approach [46] against the human reference proteome sequence database (20,435 entries, 26/09/2024) with the inclusion of common contaminants (381 entries [47]). The search criteria were trypsin cleavage of arginine and lysine (except when followed by proline), allowing for up to two missed cleavages for peptide lengths of 7 to 52 amino acids. Carbamidomethyl modification of cysteine was set as a fixed modification and protein N-terminal acetylation and methionine oxidation as variable modifications. Only proteins detected with more than one unique peptide, excluding contaminants, were included for downstream analysis. Normalized protein intensity values from Spectronaut were log_2_-transformed prior to statistical analysis. Zero intensities were handled as missing values and no values were imputed. Consistent intensity levels were observed across this series of sample measurements, indicating low variability (Additional file 2: Fig. S2). Additional assessment of the technical variation was made from the replicate analyses of the QC sample, indicating a median coefficient of variation of 15% (Additional file 2: Fig. S3). PCA analysis of proteomics data did not reveal any separation of the samples based on the available patient characteristics. Consequently, the patient ID was the only covariate used to account for variability between patients.

Differentially expressed proteins were identified using the reproducibility-optimized test statistic (ROTS v1.34.0) [48] with default parameters. The maximum top list size K was set to 75% of all proteins, and the seed was set to 1. For the comparison between gluten-free diet and post-gluten challenge samples from the same placebo-treated individuals (PGCp vs. GFDp), ROTS was applied in paired mode. The comparison between post-gluten challenge samples of drug-treated and placebo-treated individuals (PGCp vs. PGCd) was performed using unpaired mode. P values were corrected for multiple testing using the Benjamini-Hochberg procedure [49]. To assess the overall similarity of expression changes between the two comparisons, log₂ fold changes of all proteins were used to determine the Pearson correlation coefficient and its statistical significance. Statistical analyses were performed using the statistical software R version 4.4.3 (R Foundation for Statistical Computing).

#### Multi-omics integration analysis

Multi-omics integration was performed using the DIABLO framework implemented in the *mixOmics* R package (v6.34.0) [50]. Matched DNA methylation, lipidomics, and proteomics datasets were analyzed from 71 samples with complete multi-omics profiles. Each dataset was aligned by sample ID and scaled to unit variance prior to modeling. Further method details can be found in the Additional file 1: Supplementary Methods.

## Results

### Plasma lipidomic profiling reveals broad structural shifts following gluten challenge

**Fig. 1:**
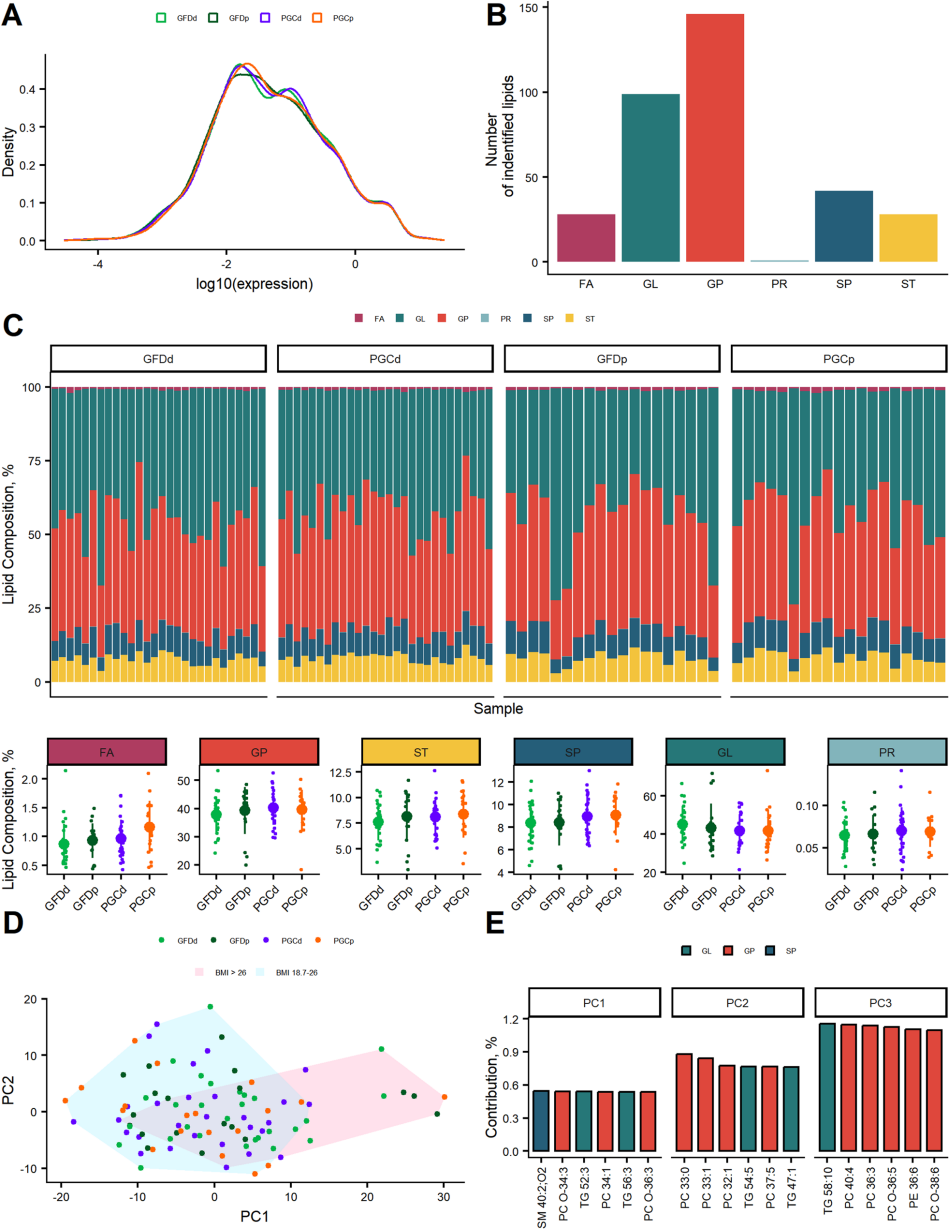
Lipidomic analysis and principal component distribution in celiac patient plasma samples.** A** The density distribution of detected lipid expression after log10 transformation reveals a uniform distribution across all four sample groups: total samples (*n* = 94), GFDd (*n* = 28), GFDp (*n* = 19), PGCd (*n* = 28), and PGCp (*n* = 19). **B** Barplot illustrates the number of identified lipids (*n* = 308) in the plasma of celiac patients, categorized by their structural type: Fatty Acyls (FA), Glycerolipids (GL), Glycerophospholipids (GP), Prenol Lipids (PR), Sphingolipids (SP) and Sterol Lipids (ST). **C** The composition of lipids by structural category is presented in barplots for individual patients (top) and collectively for patient groups (bottom diagram, mean ± SD). GP, GL and SP were identified as the primary lipid components in plasma. **D** Principal Component Analysis (PCA) scores plot demonstrates that the first and second principal components (PC1 and PC2) account for 32.7% and 11.1% of the variance, respectively. Sample groups, including GFDd, GFDp, PGCd, and PGCp, are represented by green, dark green, blue, and orange circles, respectively. Shaded areas in pink and blue represent samples with BMI values greater than 26 and between 18.7 and 26, respectively. Although there’s no clear separation of the samples by groups, there’s a notable correlation between PC1 and BMI. **E** This panel highlights the top contributors to PC1, PC2, and PC3. A majority of these contributors belong to the GP and GL categories

To characterize lipidomic alterations induced by gluten exposure, plasma lipid profiles were measured in CeD patients receiving placebo or ZED1227 after 6 weeks of gluten challenge (PGC) (Additional file 2: Fig. S1). A total of 308 lipid species, spanning six structural categories, were identified and quantified. Log-transformed intensity distributions were consistent across all sample groups, indicating high data quality (Fig. 1A, B).

Lipid composition was further examined at the structural category level. Considerable inter-individual variability was observed across lipid classes, with no extreme outliers detected (Fig. 1C). The major plasma lipid categories were glycerophospholipids (GP), glycerolipids (GL), and sphingolipids (SP). Although minor differences in class proportions were observed before and after gluten challenge, group-level mean values were largely comparable and no statistically significant changes in lipid class composition were detected between time points or treatment groups (Fig. 1C).

On principal-component analysis (PCA) there was no difference between groups based on diet or treatment. However, stratification by body mass index (BMI) revealed a clear separation, with individuals having a BMI > 26 clustering distinctly along PC1 (Fig. 1D). PC1 accounted from 32.7% of the total variance and was significantly associated with BMI (*p* < 0.001). The top contributors to PC1 are shown in Fig. 1E, with the majority of lipids belonging to the GP and GL class. When compared to BMI, the trend was consistent in both the GFDp and PGCp groups (Additional file 2: Fig. S4), indicating that the BMI-lipidome relationship was maintained regardless of gluten exposure. PC2 explained 11.1% of the total variance but was not significantly associated with any of the examined demographic or clinical variables, including BMI, sex, age, or country (Table 3). This component therefore appears to reflect inter-individual variability in lipid composition not explained by the measured covariates. Additionally, PC3 was significantly correlated with sex and country of residency during the drug treatment, while no significant correlations were observed between the first three principal components and age (Table 3). Exploratory PCA analyses annotated by selected concomitant medications did not reveal evident clustering or separation associated with medication use (Additional file 2: Fig. S5) listed in Table 2. Exploratory UMAP analysis showed a similar overall structure to PCA, with no clear separation according to treatment or diet group (Additional file 2: Fig. S6).

**Table 3:** P values for the regression of principal components against various demographic and laboratory parameters variables

Variable	PC1	PC2	PC3
Sex	0.66	0.44	**0.04**
Age	0.07	0.09	0.08
BMI	**< 0.001**	0.94	0.32
Country	0.60	0.16	**0.04**

PC1, PC2, and PC3 accounted for 32.7%, 11.1%, and 7.3% of the variability, respectively, in the current data set

Significant correlations are shown in bold

### Gluten challenge induced differential lipid expression is attenuated by ZED1227 treatment

**Fig. 2:**
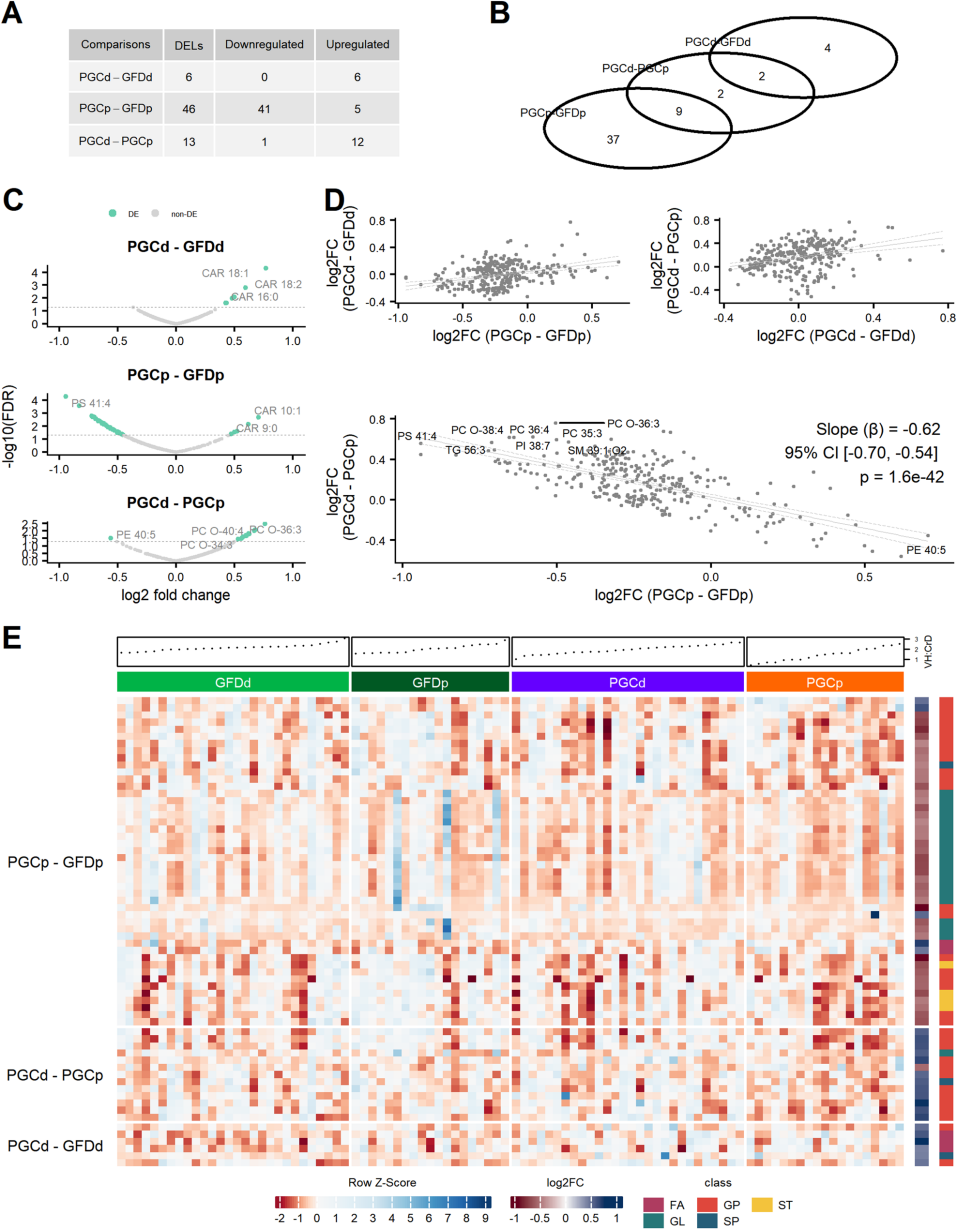
Changes of plasma lipidomic profiles of celiac patients after gluten challenge combined with placebo or ZED1227 treatment.** A** Table showing the number of differentially expressed lipids (DELs) in the indicated comparisons: 6 for PGCd vs. GFDd, 46 for PGCp vs. GFDp, 13 for PGCp –vs PGCd; total samples (*n* = 94), GFDd (*n* = 28), GFDp (*n* = 19), PGCd (*n* = 28), and PGCp (*n* = 19). **B** Venn diagram illustrating the number of DELs that are shared in the comparison of PGCp vs. PGCd and PGCp vs. GFDp (9 DELs); PGCp vs. PGCd and PGCd vs. GFDd (2 DELs). **C** Volcano plot representations comparing DELs. Green dots indicate statistically significant DELs with a p-value ≤ 0.05. The dashed horizontal line represents the p-value threshold. CAR - fatty acyl carnitines; CAR 18:2 – octadecadienylcarnitine; CAR 18:1 – octadecenoylcarnitine; CAR 16:0 – palmitoylcarnitine; CAR 10:1–9-decenoylcarnitine; CAR 9:0 – nonanoylcarnitine; PS – diacylglycerophosphoserines; PE – diacylglycerophosphoethanolamines; PE 40:5 - PE(18:1e/22:4), PC O- -glycerophosphocholines. **D** Correlation profiles of all identified lipids (*n* = 308) log2FC between PGCp vs. GFDp and PGCd vs. GFDd, PGCd vs. GFDd and PGCd vs. PGCp show consistent directionality of lipid expression changes. In contrast, the PGCp vs. GFDp and PGCd vs. PGCp correlations show opposite directionality of lipid expression. Solid lines indicate linear regression fits and dashed lines show 95% confidence intervals. The inverse association is supported by a significant negative slope (β = −0.62, 95% CI − 0.70 to − 0.54, *p* = 1.6 × 10⁻⁴²). **E** Heatmap depicting the expression of all detected DELs in tested comparisons across all samples. Lipids in rows are grouped by group comparisons, and samples are in ranking order of increase in histological villus height vs. crypt depth (VH: CrD), as depicted in the charts above the heatmaps. DELs are categorized by their structural type: Fatty Acyls (FA), Glycerophospholipids (GP), Sterol Lipids (ST), Glycerolipids (GL), and Sphingolipids (SP)

To identify lipids significantly altered by gluten exposure and drug treatment, we performed differential expression analysis with patient characteristics such as age and sex. Considering the influence of patient characteristics on PC analyses (Fig. 1D; Table 3), these variables were included as covariates in the fitted model to determine differential lipid expression. Although several lipids reached nominal significance (*p* < 0.05), none survived false discovery rate correction for multiple testing, consistent with the limited statistical power of the study. These results should therefore be interpreted as exploratory. The greatest number of differentially expressed lipids (DELs, *n* = 46) was detected in the PGCp vs. GFDp comparison (Fig. 2A), the two most abundant structural categories being GP (21 DELs) and GL (18 DELs) (Fig. 2E and Additional file 3: Table S4). Among the 46 DELs, the findings showed upregulation of two medium-chain acylcarnitines- CAR 10:1 (9-decenoylcarnitine) and CAR 9:0 (Nonanoylcarnitine) (Fig. 2C, middle panel).

When comparing the PGCd vs. GFDd, only 6 DELs were found, with no overlap with the DELs in the PGCp vs. GFDp comparison (Fig. 2B), which may be consistent with a potential attenuation of gluten-related effects, although this observation did not reach statistical significance after multiple-testing correction. In the PGCd vs. GFDd comparison, lipids belonging to 3 structural categories were detected – FA (3 DELs), GP (2 DELs), and SP (1 DEL) (Fig. 2E and Additional file 3: Table S4). Notably, half (or 25% of all identified carnitines) of the DELs are long-chain carnitines: CAR 18:2, CAR 18:1, and CAR 16:0 (Fig. 2C, upper panel).

In the PGCd vs. PGCp comparison, lipids from three structural categories were detected – GP (11 DELs, ), GL (1 DEL), and SP (1 DEL), including nine that overlapped with the PGCp vs. GFDp comparison (Fig. 2B and C, and Additional file 3: Table S4). The nine nominally significant lipids showed opposite directions of change between the two comparisons (Fig. 2D, upper panel). When all detected lipids were included (Fig. 2D, lower panel), a strong inverse relationship was observed between the lipid changes induced by gluten challenge in the placebo group (PGCp vs. GFDp) and the between-group difference after treatment (PGCd vs. PGCp). Linear regression analysis demonstrated a significant negative slope (β = −0.62, 95% CI − 0.70 to − 0.54, *p* = 1.6 × 10⁻⁴²), indicating that lipids altered by gluten challenge tended to change in the opposite direction in the drug-treated group. This pattern is consistent with attenuation of gluten-induced lipidomic changes by ZED1227.

### ZED1227 inhibits gluten-induced alterations in systemic plasma proteomic profiles and blood cell DNA methylation landscapes

**Fig. 3:**
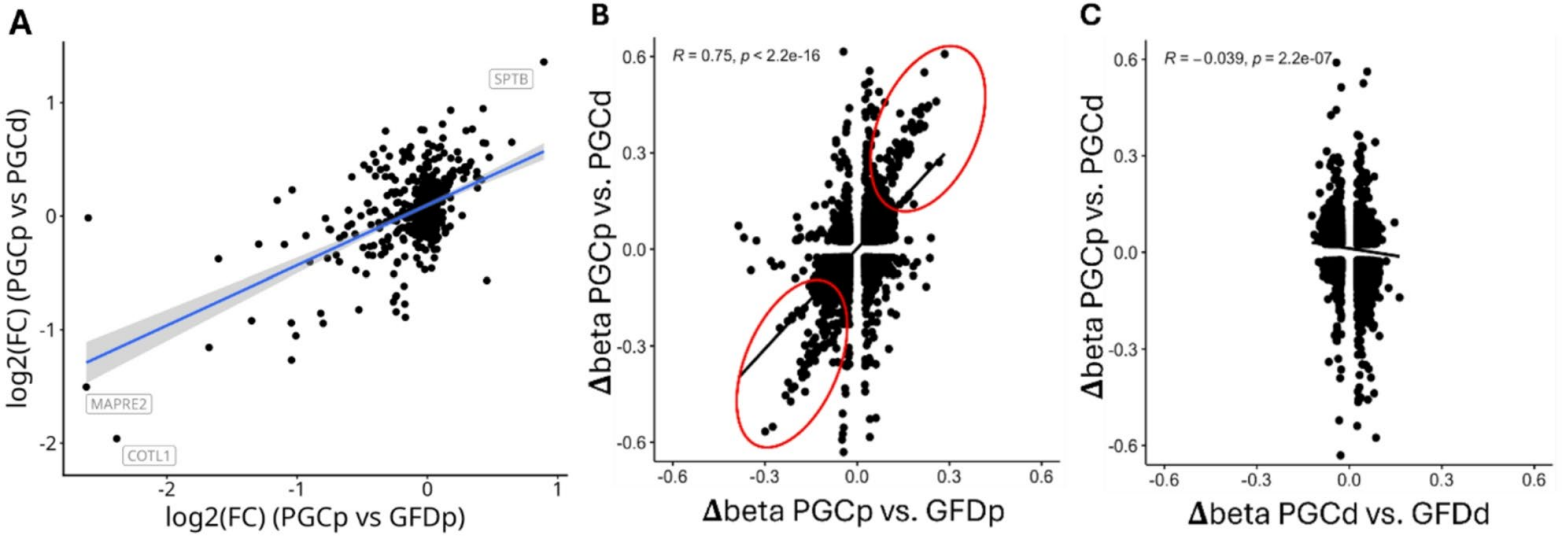
Systemic proteomic and epigenetic restoration with ZED1227. **A** Correlation between log_2_ fold changes of comparisons PGCp vs. GFDp and PGCp vs. PGCd in the proteomics data (Pearson correlation coefficient 0.55, *p* < 10^− 15^). Each data point represents a protein (*n* = 512) and proteins with largest fold changes are labelled with their corresponding gene name.Shading indicates the 95% confidence interval of the fitted regression line. **B** CpG sites having changes in methylation more than 0.025 beta units (Δβ) are shown. Red circles indicate the strong correlation in comparison between intraindividual Δβ’s in PGCp vs. GFDp and interindividual Δβ’s in PGCp vs. PGCd. **C** No such correlation was detected when intraindividual Δβ’s in PGCd vs. GFDd was compared to interindividual Δβ’s in PGCp vs. PGCd. Spearman correlation analysis was conducted to assess CpG sites exhibiting a change in beta values greater than 2.5% in both the drug and placebo conditions across different timepoints, as well as between treatment groups at the challenge phase

To further investigate biochemical changes associated with the ZED1227 treatment, plasma proteomics analyses were made. For these analyses, an approach based on the direct analysis of the digests was selected to avoid the biases and technical variability imposed by depletion and other processing strategies [51, 52]. Combining DIA mass spectrometry with the robust Evosep chromatographic instrumentation enabled detection and quantification of 512 proteins with 439 +/- 67 proteins detected per sample (Additional file 4: Table S5). Differential protein expression was tested between baseline and gluten exposure among placebo-treated patients, PGCp vs. GFDp, as well as post-exposure between drug- and placebo-treated, PGCp vs. PGCd, patients. After multiple testing corrections, no protein reached statistical significance in either of the two comparisons (adjusted *p* > 0.05, data not shown). However, when the expression changes of all proteins from both comparisons were assessed, a positive correlation of 0.55 was observed (*p* < 10^− 15^, Fig. 3A). This could be an indication that treatment with ZED1227 might modulate the patient’s plasma proteome in a manner similar to a gluten-free diet, when compared to gluten-challenged state under placebo treatment.

Given the observed changes in both lipidomic and proteomic analysis, we investigated whether these were accompanied by epigenetic modifications via DNA methylation. DNA methylation analysis of approximately 800,000 CpG sites from peripheral blood revealed no statistically significant differences (FDR < 0.05) between baseline and gluten exposure in the full cohort, or within placebo- or drug-treated subgroups. To explore broader methylation trends beyond stringent significance thresholds, CpG sites exhibiting an absolute change in methylation (Δβ) greater than 0.025 were examined, independent of p-value. This approach identified a subset of CpG sites displaying concordant methylation shifts in both the placebo group (GFD vs. PGC) and between PGC groups (placebo vs. ZED1227). The magnitude of methylation change at these CpG sites was strongly correlated between the two comparisons (Pearson’s *R* = 0.75), suggesting that gluten-induced epigenetic alteration in the placebo group is partially reversed or attenuated in the presence of ZED1227 (Fig. 3B). In contrast, no such relationship was observed when comparing the placebo vs. drug treated groups at PGC with the GFD vs. the drug-treated PGC group, which showed only a weak negative correlation with *R* = -0.039 (Fig. 3C). These findings suggest that ZED1227 may dampen systemic epigenetic responses to gluten challenge, particularly at CpG sites altered in untreated individuals.

### Carnitine-associated lipid profiles correlate with kidney function during gluten challenge and TG2 inhibitor treatment

**Fig. 4:**
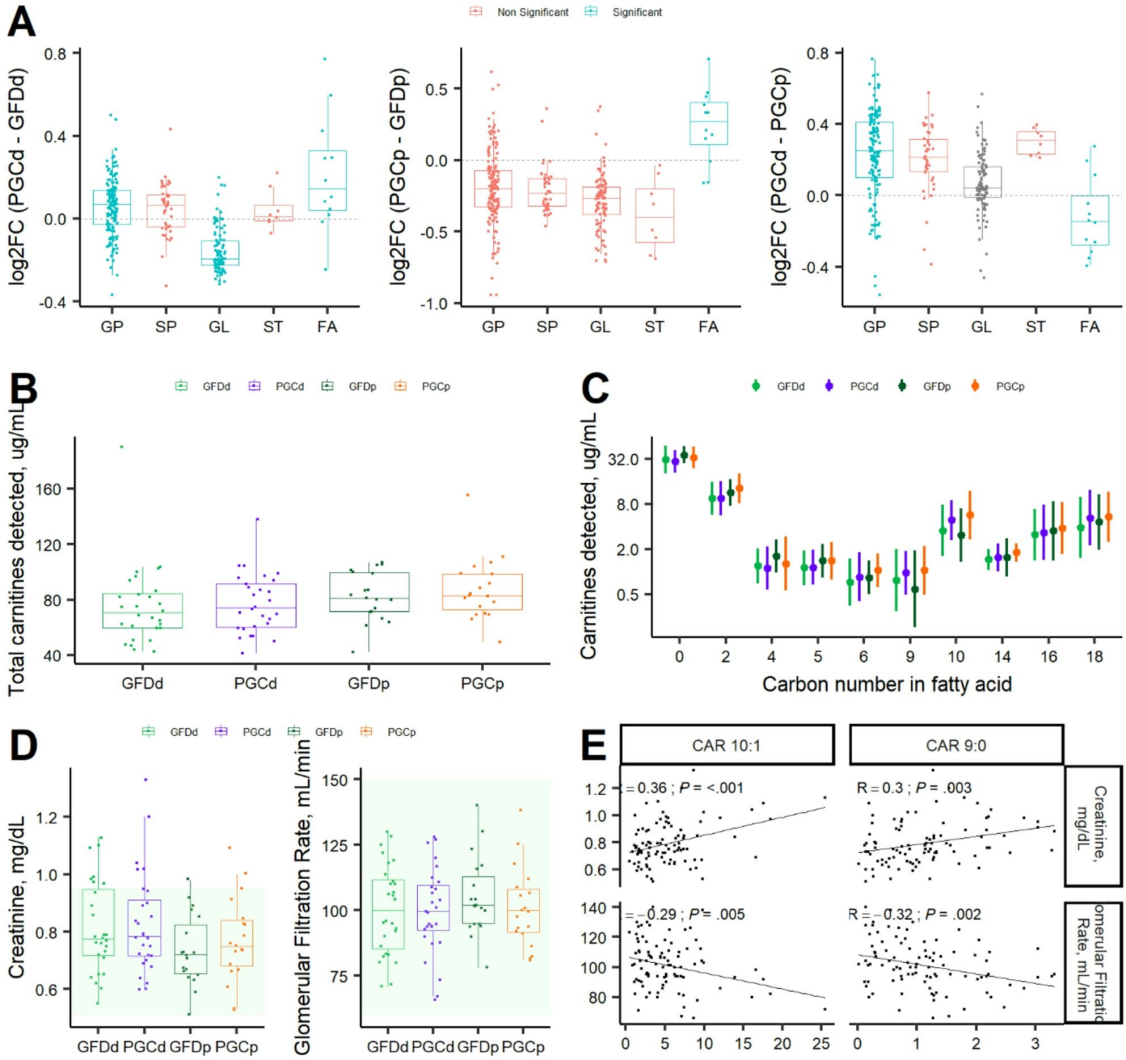
CAR 10:1 and CAR 9:0 correlate with indicators of kidney functions in celiac patients before and after the gluten challenge. **A** Enrichment analysis. Lipids were ranked by their fold change and then grouped by their structural categories. Fatty acyls (FA, consisting of fatty acyl carnitines) and glycerophospholipids (GP) were over-represented in both PGCp and PGCd groups. Fatty acyls (FA), glycerophospholipids (GP), sterol lipids (ST), sphingolipids (SP), glycerolipids (GL), and prenol lipids (PR). **B** Expression of total carnitines detected in the plasma of celiac patients, divided by groups: total samples (n = 94), GFDd (n = 28), GFDp (n = 19), PGCd (n = 28), and PGCp (n = 19). **C** Carnitines are expressed by the length of their fatty acid. Fatty acylcarnitines with chain lengths 10 and 9 were differentially expressed in the PGCp-GFDp comparison. **D** Levels of Creatinine in mg/dL in plasma and the calculated Glomerular filtration rate in mL/min for celiac patients. The green shading indicates the normal range for these laboratory tests (0.51–0.95 mg/dL for plasma Creatinine and 60–150 mL/min for glomerular filtration Rate). The figure includes the results of a two-sided paired t-test for p-value calculations, highlighting statistically significant findings (P < 0.05). **E** The correlation of plasma creatinine and glomerular filtration rate indicators with CAR 10:1 and CAR 9:0 is statistically significant. Pearson correlation coefficient is shown, and P-values less than 0.05 are considered to be significant. CAR 10:1–9-decenoylcarnitine; CAR 9:0 – Nonanoylcarnitine

Enrichment analysis of lipid categories revealed fatty acyls (FA) consisting of fatty acyl carnitines (CARs) as a key group affected by both gluten and drug treatment. FAs were upregulated in PGCp vs. GFDp and PGCd vs. GFDd, and downregulated in PGCd vs. PGCp, highlighting their role as markers of metabolic response (Fig. 4A). Notably, long-chain CARs, including CAR 18:2, CAR 18:1, and CAR 16:0 were upregulated in the PGCd in comparison with its baseline (GFDd) while medium-chain CARs (CAR 10:2 and CAR 9:0) increased in PGCp (Fig. 2C). However, the total concentration of CARs detected did not appear to differ among the patient groups (Fig. 4B). When CARs were categorized by the length of their fatty acid chains the expression levels of all lengths were comparable, except for L-carnitine (0) and acetylcarnitine (2) (Fig. 4C).

While the differential expression of CARs provides insight into metabolic responses to treatment, carnitine blood levels have also been shown to have variation in different diseases, such as liver cirrhosis of various etiologies [53, 54], renal disease [55], Crohn’s disease [56], and CeD [57]. In order to understand the clinical implications of these findings, we checked routine laboratory test results available for our study cohort.

To assess the clinical significance of CAR alteration, we analysed kidney function, as indicated by plasma creatinine levels and glomerular filtration rate (GFR). Patients in the placebo group exhibited a tendency towards decreased kidney function, as indicated by increase in plasma creatinine (from 0.73 ± 0.12 to 0.77 ± 0.15 mg/dL, *P* = 0.01) and a decrease in GFR (from 105.1 ± 14.8 to 100.8 ± 15.1 mL/min, *P* = 0.006) following the 6-week gluten challenge, although values remained within normal ranges (Fig. 4D). In contrast, no significant kidney function changes were observed in the drug-treated group (PGCd) with no significant reduction in kidney function (GFR mean ± SD in mL/min: at GFDd 100.3 ± 17.1, at PGCd 99.6 ± 16.5, *P* = 0.72; creatinine mean ± SD in mg/dL: at GFDd 0.81 ± 0.16, at PGCd 0.83 ± 0.18, *P* = 0.36) (Fig. 4D). Consistently, at follow-up up to week 10 after gluten challenge and treatment with 100 mg ZED1227, no significant decline in kidney function was observed (GFR mean ± SD: 97.4 ± 16.0 mL/min) (Table 4).

**Table 4:** Renal function parameters during the study

Creatinine, mg/dL	Drug group (*n* = 28)	Placebo group (*n* = 19)
baseline	0.81 ± 0.16	0.73 ± 0.12
challenge	0.83 ± 0.18	0.77 ± 0.15
Follow-up at 10 week	0.84 ± 0.17	0.78 ± 0.14
**Glomerular filtration rate**,** mL/min**		
baseline	100 ± 17.1	105.0 ± 14.8
challenge	99.6 ± 16.5	101 ± 15.1
Follow-up at 10 week	97.6 ± 16.0	99.8 ± 16.9

Serum creatinine and estimated glomerular filtration rate (eGFR) are shown for the drug (ZED1227) and placebo groups at baseline, during the gluten challenge, and at follow-up (10 weeks). Values are presented as mean ± standard deviation

Correlation analysis demonstrated that CAR 10:1 and CAR 9:0 negatively correlated with GFR (*R* = -0.29, *P* = 0.005 and *R* = -0.32, *P* = 0.002, respectively) and positively with plasma creatinine levels (*R* = 0.36, *P* < 0.001 and *R* = 0.30, *P* = 0.003, respectively) (Fig. 4E), reinforcing their potential role as metabolic indicators of gluten-induced renal stress. CARs identified as DELs in the comparison between PGCd and GFDd groups showed no significant correlation with plasma creatinine levels, with the exceptions being CAR 16:0 and CAR 18:1, which were found to correlate with GFR (Additional file 2: Fig. S7).

### Multi-omics integration reveals coordinated immune–epigenetic–lipid remodeling

To examine coordinated molecular variation across datasets, we applied DIABLO to integrate 31,067 CpG sites, 308 lipid species, and 521 protein features. The final sparse block PLS-DA model contained two latent components selected using cross-validated balanced error rate.

**Fig. 5:**
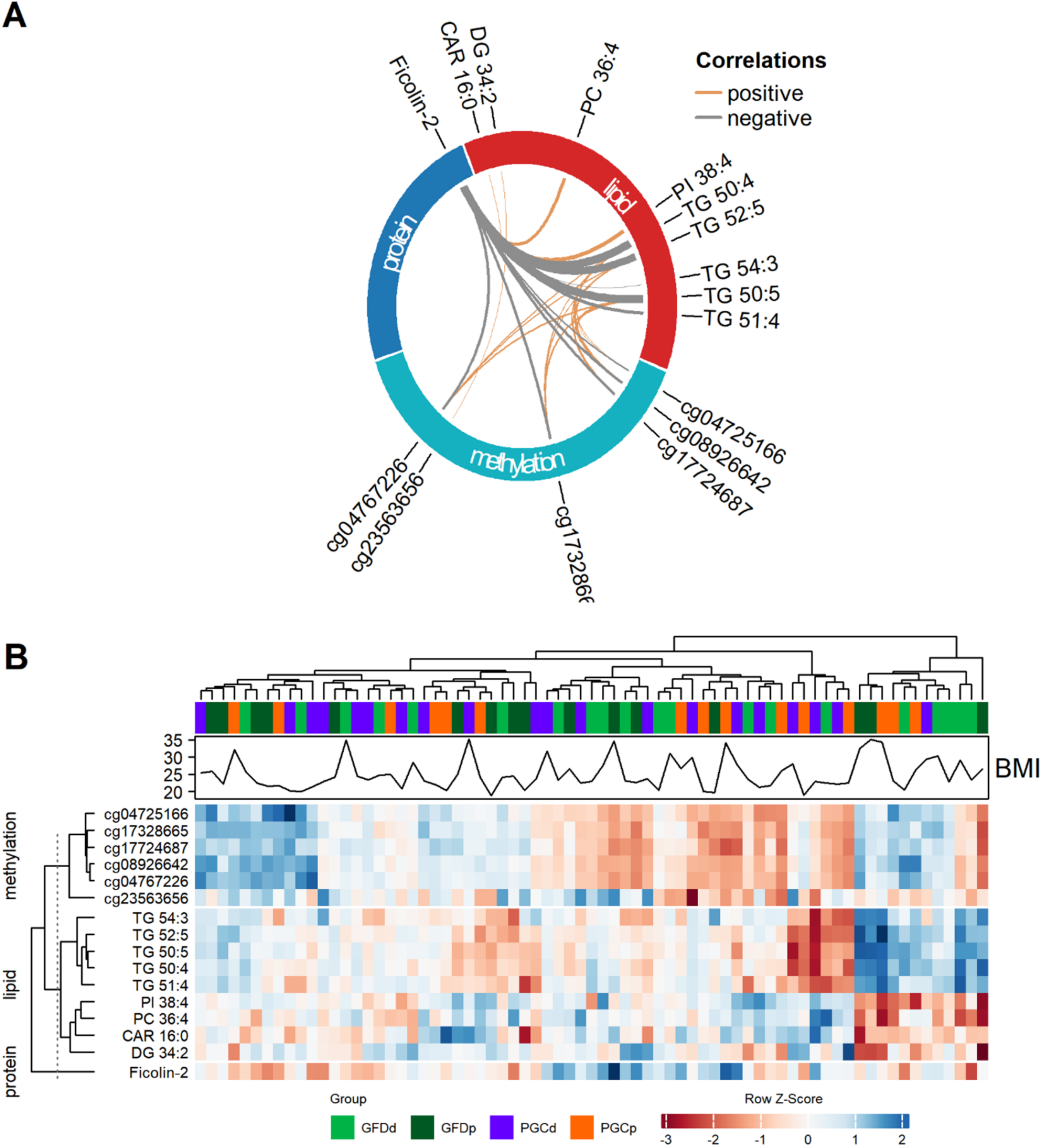
Integrated multi-omics signature associated with disease status. Multi-omics integration was performed using sparse block PLS-DA (DIABLO) on DNA methylation (31,067 CpGs), lipidomics (308 species), and proteomics (521 features). The final model included two latent components selected based on cross-validated balanced error rate. **A** Circos plot showing correlations between selected features across omics layers. Only correlations with an absolute value of |r| ≥ 0.65 are displayed. Line thickness reflects correlation strength; orange lines indicate positive correlations and grey/brown lines indicate negative correlations. **B** Heatmap of selected features illustrating coordinated variation across samples. Features cluster into modules linking *PER3*-associated CpG sites, lipid species, and protein expression. No clear association with BMI or patient group is observed

The analysis uncovered a coherent molecular module linking DNA methylation, lipid metabolism, and protein abundance (Fig. 5A). Correlation filtering (|r| ≥ 0.65) revealed a structured network centered on a protein–lipid–methylation axis. Five tightly correlated CpG sites (cg04725166, cg08926642, cg17724687, cg17328665, cg04767226) (Additional file 2: Table S6) mapped to exon 18 of *PER3* within a CpG island, forming a coordinated epigenetic cluster strongly connected to lipid and protein features. In contrast, cg23563656 in the *PPM1H* promoter (CpG shore) showed a distinct correlation pattern, indicating locus-specific regulation.

The integrated network revealed both positive and negative lipid–protein relationships. Phospholipid (PC 36:4, PI 38:4) and diacylglycerol (DG 34:2) species showed coordinated associations with methylation changes, whereas polyunsaturated fatty acids (PUFA)-enriched triglycerides (e.g., TG 50:4, TG 50:5, TG 51:4, TG 52:5) showed strong inverse associations with Ficolin-2 (Q15485), which emerged as a central hub, whereas monounsaturated TG 54:3 exhibited much weaker correlation. This indicates that the observed inverse relationship is specific to PUFA-rich triglycerides rather than total triglycerides.

Overall, the results highlight coordinated immune–metabolic interactions involving Ficolin-2, *PER3* methylation, and selective triglyceride remodeling. Despite strong internal network correlations, these features were not significantly associated with patient group or BMI (Fig. 5B), suggesting that the module reflects intrinsic biological variation rather than clinical stratification.

## Discussion

In celiac disease, repeated daily gluten exposure elicits a recall immune response that disrupts intestinal homeostasis and progressively leads to quantitatively measurable duodenal mucosal injury. This immune activation can result in systemic changes, including alternations in the plasma lipid profile detectable in peripheral blood. Lipidomic analyses have shown that patients with active CeD exhibit altered serum or plasma lipid profiles, with changes in levels of glycolipids (GLs), in particular triglycerides (TGs) [34] and diacylglycerols (DGs) [58], phosphatidylcholines (PCs), [33, 34] cholesterol derivatives, [34] and fatty acyls (FA) [58]. Such alterations are already evident in infants even before gluten exposure, for example, changes in phospholipids levels [33] and upregulation of triacylglycerols. [34] Some studies detected no differences in systematic levels of fatty acids in newly diagnosed CeD [59]. However, there are no published results on the lipidomic changes in adult CeD patients post-gluten challenge, or with/without effective drug therapy.

In the present study, we have observed distinct plasma lipidome profiles in CeD patients who underwent a gluten challenge, with and without the TG2 inhibitor ZED1227. Notable differences emerged in both cohorts exposed to gluten challenge, corresponding to the assigned intervention of placebo or the investigational TG2 inhibitor ZED1227. Out of a total of 1004 lipids detected, 308 were successfully identified. Among these identified lipids, 46, accounting for approximately 15%, demonstrated differential expression in the placebo group following a gluten challenge. Specifically, 41 lipids decreased, while 5 increased. Notably, the fatty acyl (FA) category was significantly enriched in comparison between the gluten challenged and placebo-treated group (PGCp) and the baseline gluten-free diet placebo group (GFDp). FAs, particularly eicosanoids, were previously found to be upregulated in the plasma of children with CeD compared to their non-celiac siblings. [58] However, another study did not observe changes in plasma fatty acids in children with newly diagnosed CeD. [59] These differing results might be attributed to methodological variances and differences in the studied cohorts, making direct comparisons with our findings difficult.

Interestingly, gluten challenged patients treated with ZED1227 showed significantly less differentially expressed lipids, as only six lipids were differentially expressed in the GFDd vs. PGCd comparison (Fig. 2A). Importantly, none of the lipid alterations evoked in gluten challenge appeared in patients treated with ZED1227 (Fig. 2B). The findings suggest that gluten challenges are associated with measurable changes in the plasma lipid profile at the cohort level and that the direction of these changes differs between CeD patients receiving ZED1227 and those receiving placebo. In particular, comparison of lipid profile across all detected species (*n* = 308) demonstrated opposing directionality beween PGCp vs. PGCd and PGCp vs. GFDp contrasts, consistent with an attenuation of gluten-associated lipidomic alternation in the ZED1227 groups (Fig. 2D). These analyses do not establish correlations between lipidomic changes and histological outcomes at the level of individual participants; thus, the observed plasma lipid alterations should be viewed as systemic correlates of gluten exposure rather than surrogate markers of mucosal injury. Because circulating lipids integrate signals from multiple tissues and metabolic pathways, the cellular origin and biological function of the observed changes cannot be determined from the current dataset. Further studies will be required to determine whether such lipidomic signatures reflect disease reactivation during sustained gluten ingestion and to clarify their source and functional relevance.Proteomics analyses did not identify individual proteins with statistically signification differential expression after correction for multiple testing. Notably, the representation of these proteomes was limited to highly and moderately abundant plasma proteins, among which no statistically significant differences were found. The implementation of currently available state-of-the-art technologies [60] would extend the protein coverage and potentially discern drug and gluten-induced differences in the plasma. Nevertheless, global expression patterns differed between the placebo and ZED1227 groups, with profiles in the ZED1227 study more closely resembling those observed in participants maintained on a gluten-free diet at baseline (Fig. 3A). These findings suggest that TG2 inhibition may modulate systemic plasma protein expression during gluten challenges, although effects on immune responses or specific metabolic pathways were not directly assessed in this study. Additionally, epigenetic profiling did not identify significant site-specific DNA methylation changes after gluten challenge or drug treatment. However, analysis of CpG sites with moderate methylation shifts indicated that gluten-induced epigenetic alterations in untreated patients were partially reversed or attenuated by ZED1227 (Fig. 3B&C). These findings highlight that TG2 inhibition reduced systemic epigenetic responses to gluten exposure, potentially contributing to its therapeutic efficacy in CeD patients.

In this study, small changes in renal-related laboratory evaluation were observed during the six-week gluten challenge, including a minor reduction in estimated GFR. These changes remained within normal ranges and were not associated with clinical apparent renal dysfunction. Differences in these results between ZED1227 and placebo groups were observed; however, given the exploratory nature of the analyses and the absence of prior evidence for gluten challenge-associated deterioration in renal function, these findings should be further investigations in future studies. In fact, after years and decades of gluten ingestion, untreated CeD has been linked in clinical and epidemiological studies to an increased risk of kidney diseases [14, 61, 62]. In the present study, renal function parameters in PGCp group were associated with plasma levels of medium-chain acylcarnitines, including CAR 9:0 and CAR 10:1. In fact, elevated plasma concentrations of pelargonylcarnitine (CAR 9:0) [63] and decanoylcarnitine (CAR 10:0) [64] have earlier been reported in patients with impaired kidney function. In the kidney, carnitine and its precursors are efficiently reabsorbed to minimize urinary loss [65]. It has been proposed that acylcarnitines may serve as metabolomic markers for chronic kidney disease [66]. Several studies have found an inverse association between long-chain acylcarnitines [64], as well as short-chain [67, 68] and medium-chain acylcarnitines [68], and reduced estimated glomerular filtration rate (GFR). Further studies are warranted to show whether these acylcarnitines could serve as early biomarkers for the systemic effects of gluten ingestion in CeD patients.

Carnitine plays a crucial role in energy metabolism by facilitating the transport of long-chain fatty acids into mitochondria for β-oxidation. Blood plasma levels of carnitine are maintained through dietary intake, endogenous synthesis, renal reabsorption, and cellular uptake [69]. CeD can lead to secondary carnitine deficiency [70]. Significantly lower serum total carnitine concentrations have been observed in patients compared to those on a gluten-free diet (GFD) and non-CeD controls [71]. Additionally, various acylcarnitines (CAR) have been reported to decrease in CeD patients on a GFD compared to healthy individuals [72], a condition often linked to intestinal inflammation and resulting malabsorption syndromes. Our study, however, did not detect deficiencies in plasma concentrations of L-carnitine and short-chain CARs (C1-C4). This could be attributed to our cohort of well-controlled celiac patients at baseline, and the relatively short duration of the gluten challenge they underwent. Furthermore, while an increase in Isobutyryl-L-carnitine (CAR 4:0) levels has been observed in the plasma of pediatric CeD patients showing disease progression [73], this trend was not evident in our study’s patients.

Our integrative analysis delineates a coordinated immune–epigenetic–lipid module centered on Ficolin-2 (FCN2), PUFA-enriched triglycerides, and a tightly co-regulated CpG cluster within *PER3*. The inverse relationship between Ficolin-2 and selected PUFA-rich TG species (but not monounsaturated TG 54:3) argues for selective immunometabolic coupling rather than a generic triglyceride effect. Given Ficolin-2’s role as a soluble pathogen recognition receptor (PRR) that triggers the lectin complement pathway, these data are consistent with a model in which innate immune surveillance interfaces with lipid remodeling during physiologic adaptation. In the context of CeD, where complement activation has been documented systemically and in the small-intestinal mucosa, Ficolin-2 may reflect lectin-pathway–linked activation potential within a broader immune–metabolic axis, though causality remains unresolved [74–76]. The *PER3* CpG island (exon 18) behaved as a coherent epigenetic unit within this network, aligning with evidence that circadian regulators shape metabolic and inflammatory programs. Such clock–metabolism–immunity crosstalk provides a plausible mechanism by which PER3-linked methylation tracks lipid remodeling and innate immune tone [77–79]. The specificity of correlations—with a locus-level signal at *PER3* and a distinct pattern at *PPM1H*—argues against global methylation drift and favors locus-specific regulation. Finally, the absence of associations with BMI or patient group implies that this module captures intrinsic biological variation rather than overt clinical stratification.

We acknowledge certain limitations of the current study. The sample size is relatively low, comprising only 47 CeD patients; however, the patients have been broadly characterized clinically, histologically and regarding laboratory parameters, which we consider adequate for analysis using robust statistical methods. Dietary intake is a major determinant of circulating lipid profiles; however, detailed dietary data were not available for this post hoc analysis. While all participants followed a long-term gluten-free diet prior to inclusion and underwent a standardized gluten challenge, individual variation in dietary composition, particularly fat intake, may represent a source of residual confounding. Even if the gluten challenge duration was relatively short—six weeks—with a daily intake of an intermediate amount of gluten (3 g vs. an average daily intake of 10–15 g in the non-celiac population), we observed significant lipidomic alterations upon gluten challenge, which were significantly attenuated with TG2 inhibitor treatment. Similar trends were also observed when monitoring proteomic and DNA methylomic changes (Fig. 3). The identified differentially expressed lipid species can provide a foundation for future research aimed at establishing novel biomarkers reflecting dietary effects of gluten. To conclude, our integrative multi-omics approach provides a holistic view of the molecular landscape in CeD patients undergoing gluten challenge and supports the notion that TG2 inhibitor treatment has also beneficial systemic effects.

## Conclusions

Gluten challenge induced measurable systemic lipidomic, proteomic, and epigenetic changes in treated celiac disease patients. These alterations were largely attenuated by pharmacological transglutaminase 2 inhibition with ZED1227, yielding molecular profiles closer to those observed during a gluten-free diet. Together, the results support a role for TG2 inhibition in dampening gluten-associated systemic immunometabolic responses in celiac disease.

## Supplementary Information

Below is the link to the electronic supplementary material.

Supplementary Material 1: Additional file 1: Supplementary Methods

Supplementary Material 2: Additional file 2: Figures S1-S7

Supplementary Material 3: Additional file 3: Table S4

Supplementary Material 4: Additional file 4: Table S5

Supplementary Material 5: Additional file 5: List of CEC-3 investigators

## Data Availability

The mass spectrometry discovery proteomics data have been deposited to the ProteomeXchange Consortium via the PRIDE [80] partner repository with the dataset identifier PXD069597. Processed DNA methylation data is available in GEO with access code GSE319777.

## Abbreviations

CeD: Celiac disease
TG2: Transglutaminase 2
GFD: Gluten-free diet
GFDd: Gluten-free diet drug group
GFDp: Gluten-free diet placebo group
PGC: Post gluten challenge
PGCd: Post gluten challenge drug group
PGCp: Post gluten challenge placebo group
VH:CrD: Villous height to crypt depth ratio
BMI: Body mass index
CAR: Fatty acylcarnitines
GFR: Glomerular filtration rate
HLA: Human leukocyte antigen
EDTA: Ethylenediaminetetraacetic acid
TG2 IgA: Anti-transglutaminase 2 immunoglobulin A antibodies
PCA: Principal-component analysis
DELs: Differentially expressed lipids
FA: Fatty Acyls
GP: Glycerophospholipids
ST: Sterol Lipids
SP: Sphingolipids
GL: Glycerolipids
PR: Prenol Lipids
PC O: Phosphatidylcholines
LPC: Lysophosphatidylcholines
LPE: Lysophosphatidylethanolamines
PC: Phosphatidylcholines
PE: Phosphatidylethanolamines
PE O: Ether-linked Phosphatidylethanolamines
PI: Phosphatidylinositols
PS: Phosphatidylserines
PUFA: Polyunsaturated Fatty Acids
sPLS: DA-Sparse Partial Least Squares Discriminant Analysis
