# Supplementary material for: Therapeutic TG2 inhibition reverses systemic multiomic dysregulation in celiac disease
Source: BMC Med. 2026 Apr 24;24:350. doi: 10.1186/s12916-026-04892-y (PMC13255354; doi:10.1186/s12916-026-04892-y)
Supplement: Supplementary file 2 — Supplementary Material 2: Additional file 2: Figures S1-S7 [file 12916_2026_4892_MOESM2_ESM.docx]

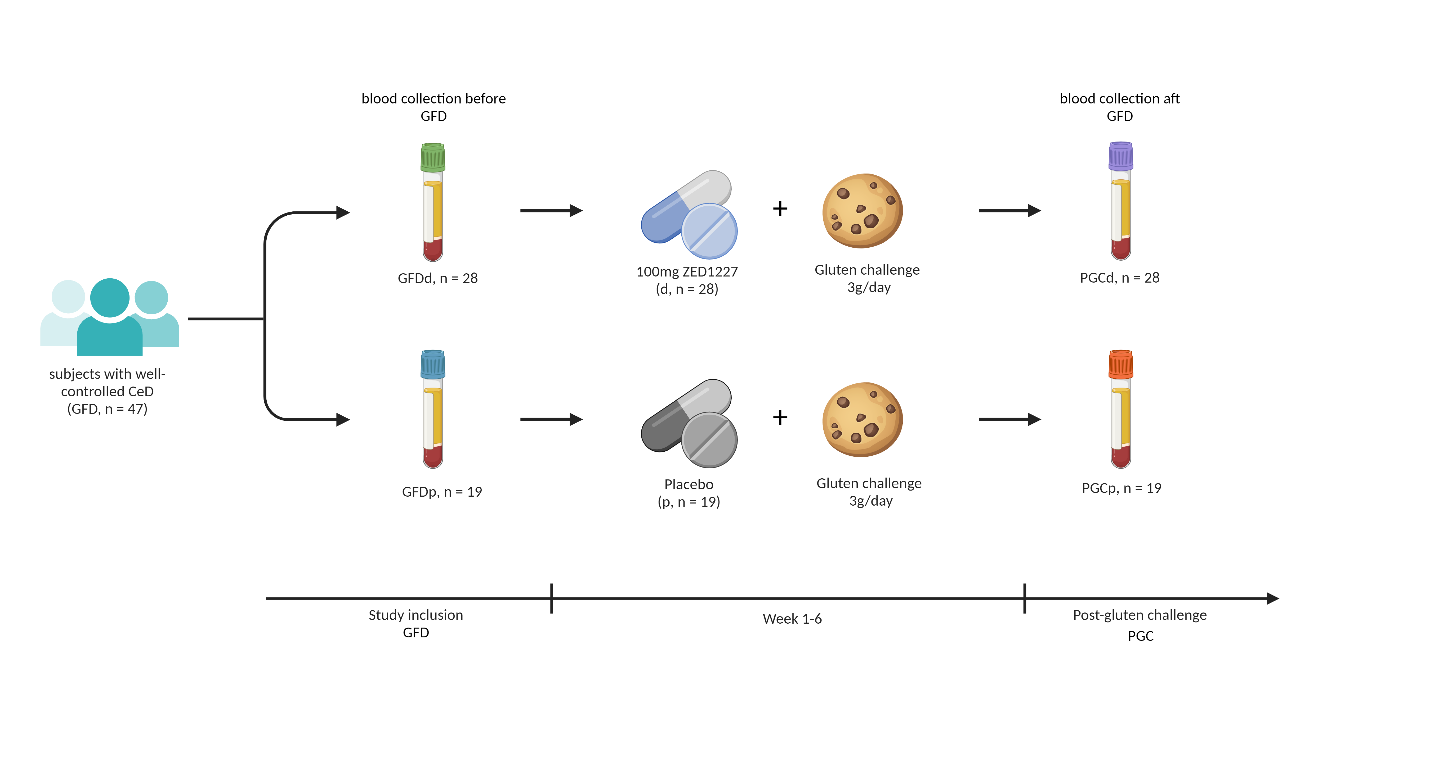
**Figure S1. Schematic presentation of the study.** Samples (n = 94; n of patients = 47), in the form of blood samples, were collected from the trial, aimed at dose-finding and assessing the efficacy and tolerability of a 6-week treatment with ZED1227 capsules vs. placebo in subjects with well-controlled celiac disease undergoing gluten challenge. Blood sampling was performed twice: on study inclusion (GFDd, n = 28; GFDp, n = 19) and at the final visit (PGCd, n = 28; PGCp, n = 19). Plasma-EDTA was separated from the cell pellet and subjected to lipidomic analysis. Created with BioRender.com.


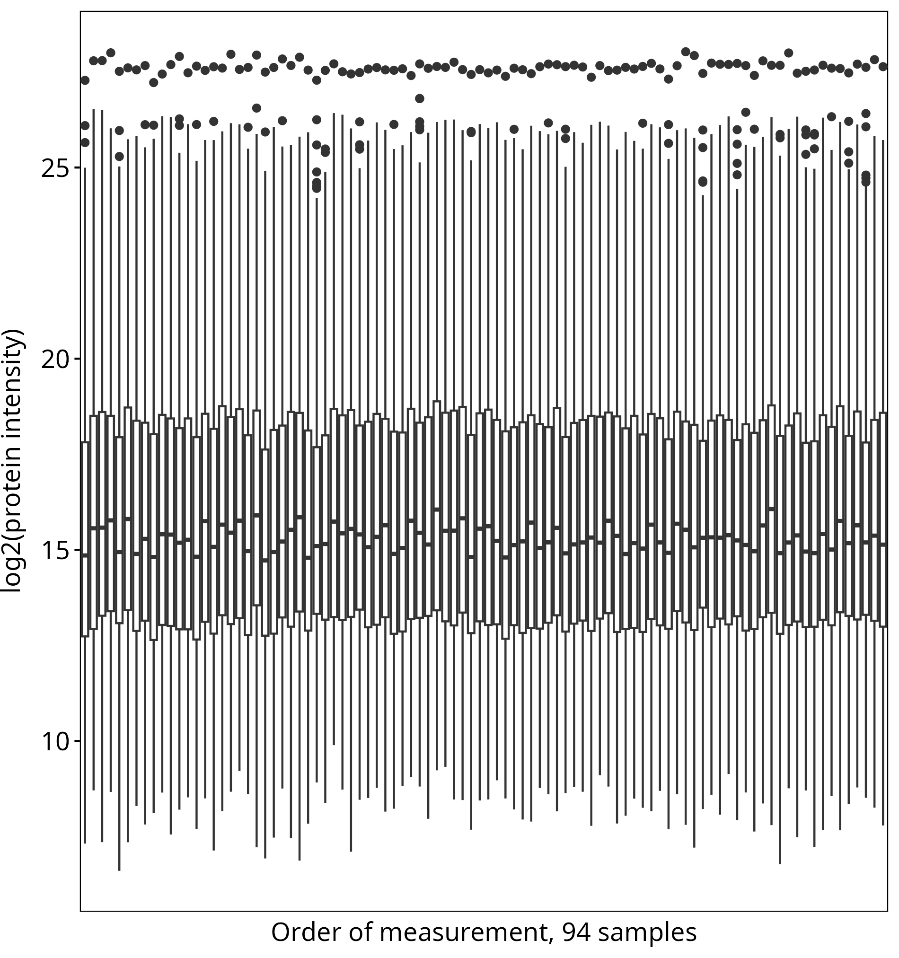


**Figure S2**. **Boxplots of intensities from all proteomics samples.** Stability of intensity levels suggests consistent measurement quality. All 94 samples were measured within 4 days. Whiskers of boxplots extend 1.5 times interquartile range from quantiles


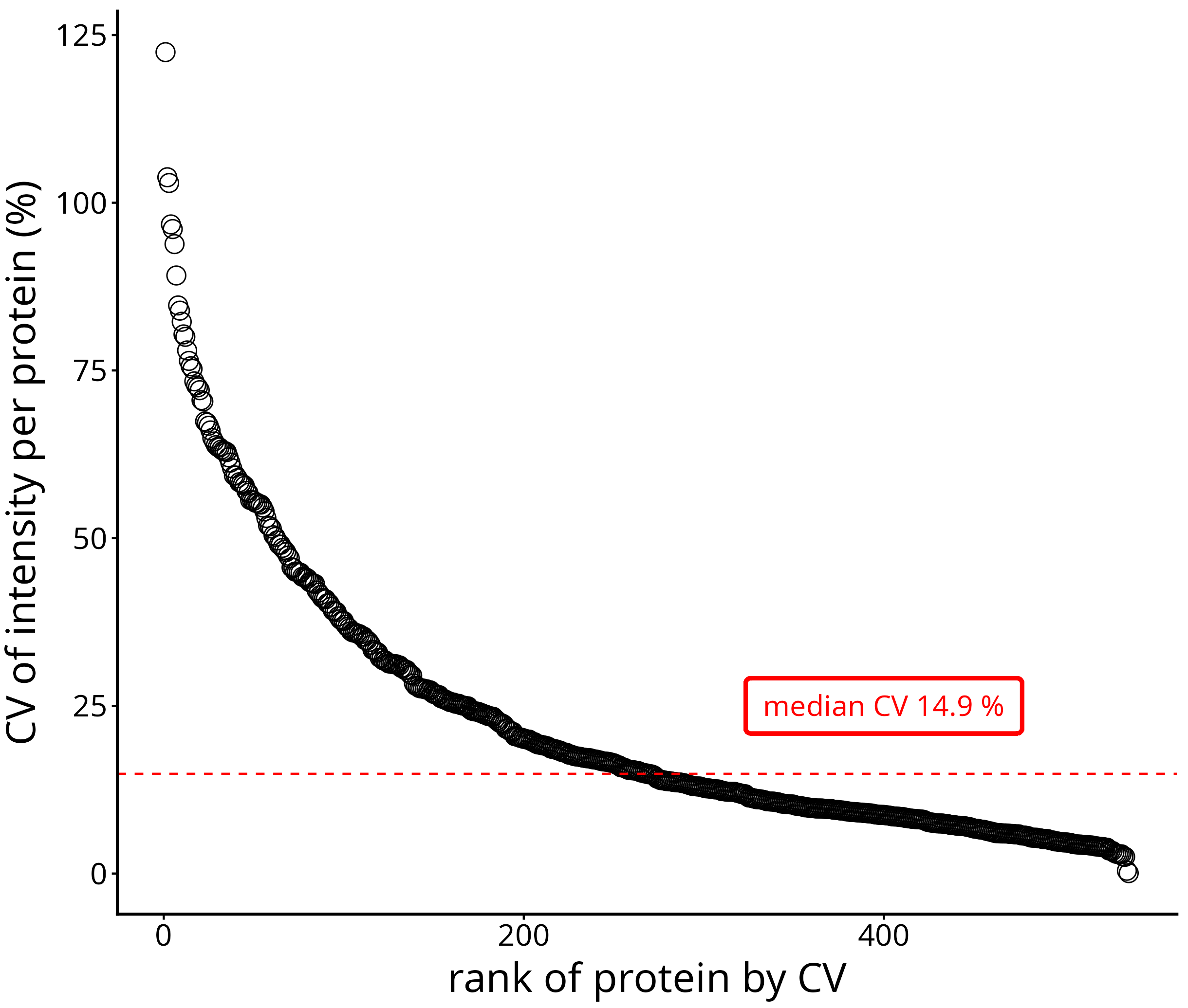


**Figure S3. Coefficients of variation (CV) for the proteins detected from the digested plasma QC sample (5 replicates).** The median of the CVs is marked with a dashed line. The CV could be calculated from a total of 536 quantified proteins.

**
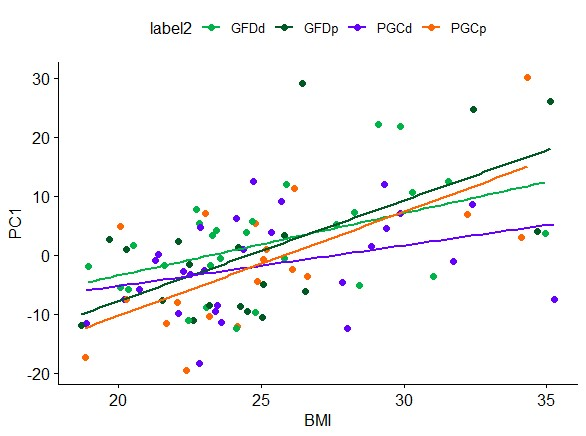
**

**Figure S4. BMI trends identified by principal component analysis (PCA). Subject** BMI was regressed against first principal component (PC1) scores separately for each group: GFDd (n = 28, green color), GFDp (n = 19, dark green color), PGCd (n = 28, violet color), and PGCp (n = 19, orange color). The lines of best fit for each group are colored to match the color of the symbol for that group. The violet line (for PGCd) seems to have a less steep slope compared to the other. The similarity in the slopes of the regressed lines indicates that BMI-related changes in lipidomic profiles were similar in each group and independent of drug treatment.


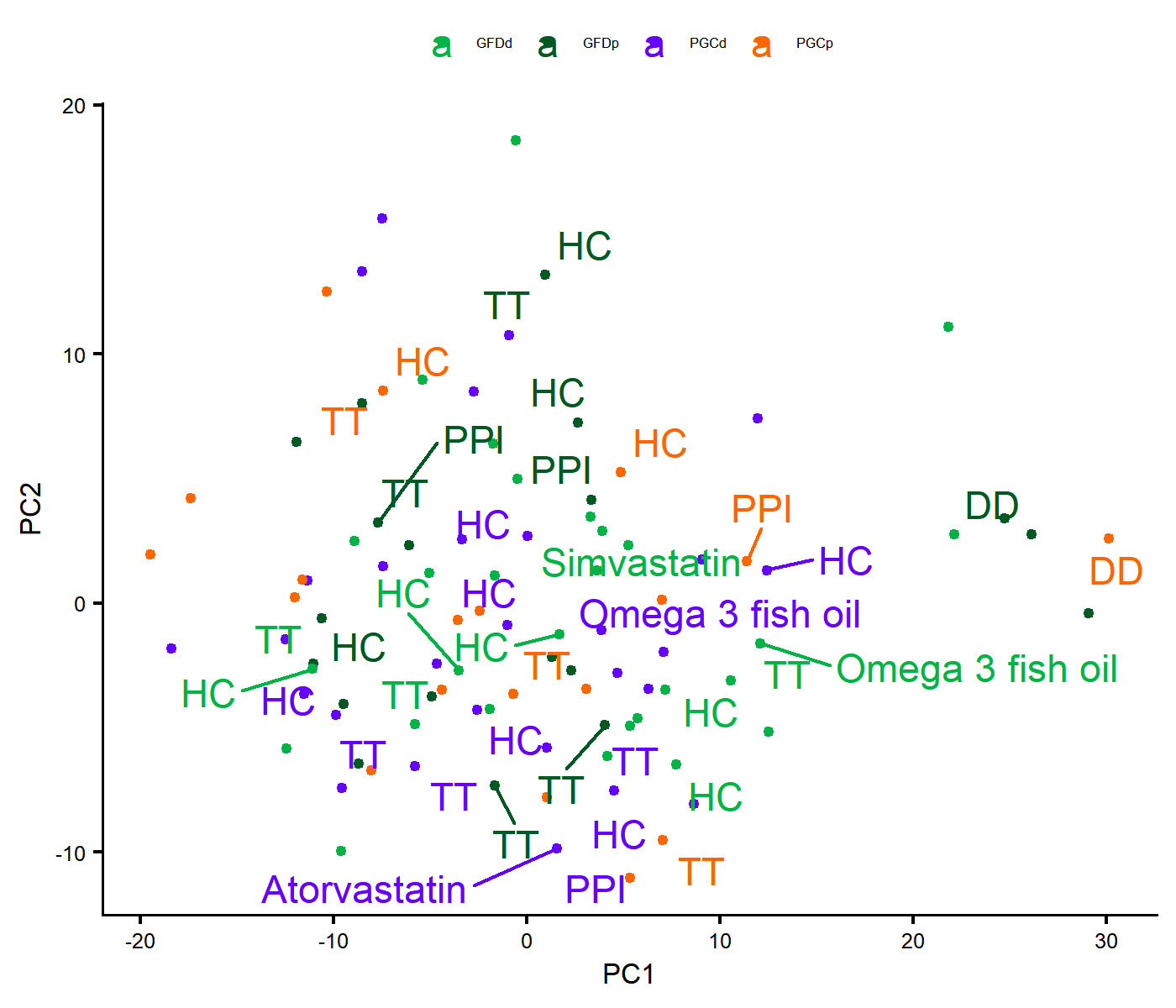


**Figure S5**. **Principal component analysis of lipidomic profiles annotated by concomitant medication use.** Individual data points represent study participants, colored by study group: GFDd (n = 28, green color), GFDp (n = 19, dark green color), PGCd (n = 28, violet color), and PGCp (n = 19, orange color). Medication labels: PPI – proton pump inhibitors, DD – Drugs used in diabetes, TT – Thyroid therapy, HC – Hormonal contraception


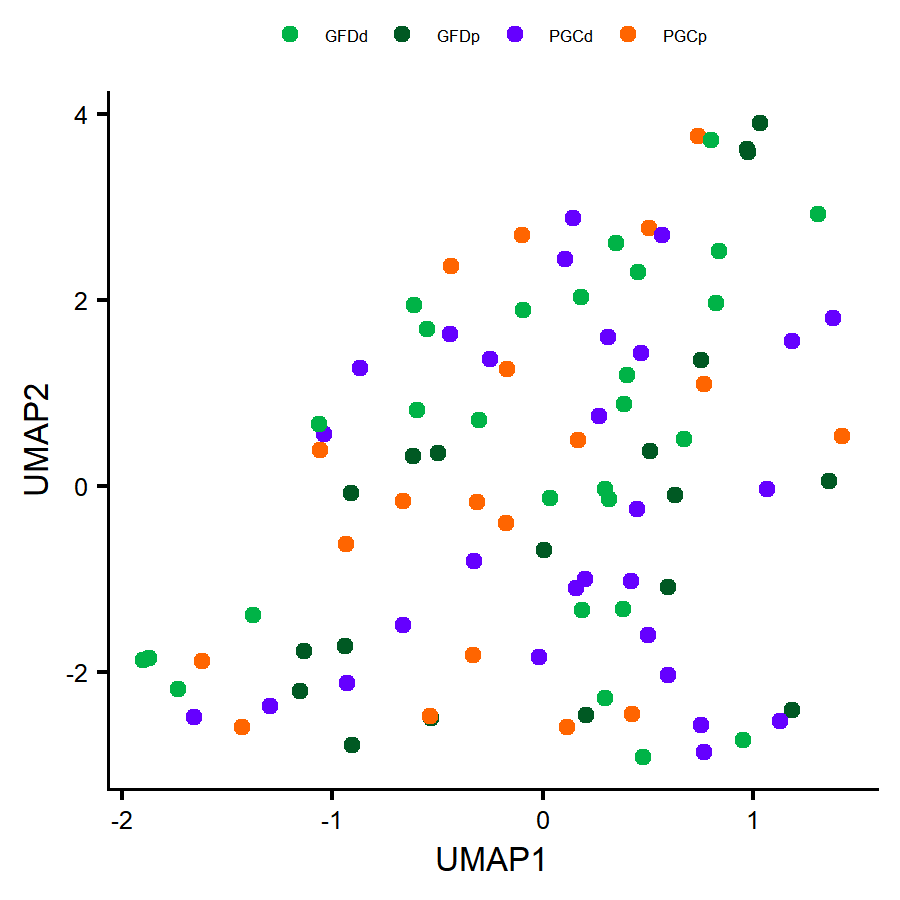


**Figure S6. UMAP visualization of lipidomic profiles across study groups.** Uniform Manifold Approximation and Projection (UMAP) was applied to the 308 identified lipid species to visualize global structure in the dataset. Each point represents one sample, colored by study group GFDd (n = 28, green color), GFDp (n = 19, dark green color), PGCd (n = 28, violet color), and PGCp (n = 19, orange color).


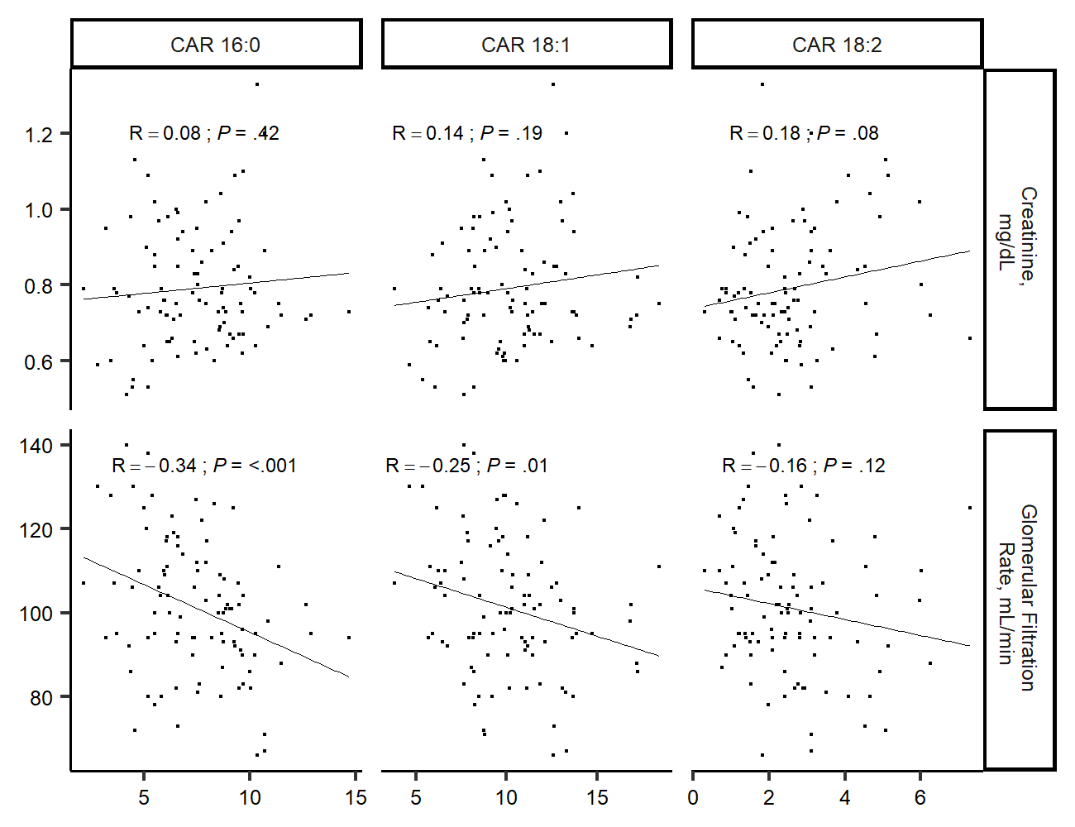


**Figure S7. Correlation of plasma Creatinine and Glomerular Filtration Rate indicators with CAR 16:0, CAR 18:1, and CAR 18:2.** Pearson correlation coefficient is shown, and P-values less than 0.05 are considered to be significant. CAR - Fatty acylcarnitines; CAR 18:2 – octadecadienylcarnitine; CAR 18:1 – octadecenoylcarnitine; CAR 16:0 – Palmitoylcarnitine.

**Table S1.** Demographic Characteristics of the Patients in original cohort and in present study.

|  | Original cohort* | | Present study cohort (Lipidomic and Proteomics) | | Present study cohort (Methylomics) | |
| --- | --- | --- | --- | --- | --- | --- |
| Characteristic | ZED1227, 100 mg (N = 39) | Placebo (N = 38) | Drug(d, n=28) | Placebo(p, n=19) | Drug(d, n=20) | Placebo(p, n=16) |
| Age — yr (mean ± sd) | 41.0±14.8 | 42.5±14.4 | 40.9 ± 15.0 | 45.3 ± 14.8 | 40.7 ± 15.1 | 46.8 ± 14.6 |
| Female sex — no. (%) | 24 (62) | 28 (74) | 18 (64.3) | 12 (63.2) | 13 (65.0) | 10 (62.5) |
| White race — no. (%) | 39 (100) | 38 (100) | 28 (100) | 19 (100) | 20 (100) | 16 (100) |
| Weight — kg (mean ± sd) | 73.2±13.7 | 68.4±14.7 | 74.5±14.5 | 71.8±17.2 | 73.1±14.9 | 72.2±18.1 |

*Original cohort Demographic Characteristics is published in Schuppan, D. et al. A Randomized Trial of a Transglutaminase 2 Inhibitor for Celiac Disease. N. Engl. J. Med. 385, 35–45 (2021).

**Table S2.** Effect of ZED1227 Treatment on the Ratio of villus height to crypt depth (VH:CrD) in original cohort and in present study.

|  | Original cohort* | | Present study cohort (Lipidomics and Proteomics) | | Present study cohort (Methylomics) | |
| --- | --- | --- | --- | --- | --- | --- |
| Variable | ZED1227, 100 mg (N = 38) | Placebo (N = 30) | Drug(d, n=28) | Placebo(p, n=19) | Drug(d, n=20) | Placebo(p, n=16) |
| VH:CrD |  |  |  |  |  |  |
| GFD | 2.09±0.35 | 1.98±0.33 | 2.12±0.33 | 2.00±0.39 | 2.10±0.28 | 2.02±0.42 |
| PGC | 1.94±0.48 | 1.39±0.61 | 1.91±0.42 | 1.41±0.64 | 2.00±0.37 | 1.47±0.62 |
| Change from GFD (95% CI) | -0.13(-0.28 to 0.03) | -0.61(-0.78 to -0.44) | -0.21(-0.38 to -0.03) | -0.60(-0.85 to -0.35) | -0.10(-0.26 to -0.06) | -0.55(-0.82 to -0.29) |

*Original cohort VH:CrD descriptive statistics is published in Schuppan, D. et al. A Randomized Trial of a Transglutaminase 2 Inhibitor for Celiac Disease. N. Engl. J. Med. 385, 35–45 (2021).

Plus–minus values are means ±SD. The change from GFD is presented as a least-squares means estimate.

**Table S3**. Summary of lipid detection and data completeness across samples

| **Metric** | **Value** |
| --- | --- |
| Total lipids detected | 1007 |
| Structurally identified lipids | 308 |
| Median identified lipids per sample | 308 |
| Range identified lipids per sample | 305-308 |

**Table S6.** Annotation of CpG sites included in integrative analysis presented in the Figure 5
