# Supplementary material for: Therapeutic TG2 inhibition reverses systemic multiomic dysregulation in celiac disease
Source: BMC Med. 2026 Apr 24;24:350. doi: 10.1186/s12916-026-04892-y (PMC13255354; doi:10.1186/s12916-026-04892-y)
Supplement: Supplementary file 5 — Supplementary Material 5: Additional file 5: List of CEC-3 investigators [file 12916_2026_4892_MOESM5_ESM.pdf]

#List of CEC-3 Trial Group Collaborators

Investigators

**Country**	**Principal Investigator**	**Institution**
Estonia	**Karin Kull, MD**	Department of Gastroenterology, Internal Medicine Clinic, Tartu University Hospital, Tartu
Finland	**Jari Koskenpato, MD**	Lääkärikeskus Aava Helsinki Kamppi, Helsinki
	**Mika Scheinin, MD, PhD**	Clinical Research Services Turku - CRST Oy, Turku
	**Marja-Leena Lähdeaho, MD, PhD***	Faculty of Medicine and Health Technology, Tampere University and Tampere University Hospital, Tampere<br>*and Department of Pediatrics, Tampere University Hospital, Tampere
Germany		
	**Michael Schumann, MD**	Department for Gastroenterology, Infectious diseases and Rheumatology, Campus Benjamin Franklin, Charité - University Medicine Berlin, Berlin
	**Yurdagül Zopf, MD**	Department of Medicine 1, Hector Center for Nutrition, Exercise, and Sports, Universitätsklinikum Erlangen, Friedrich-Alexander-University Erlangen-Nürnberg, Erlangen
	**Andreas Stallmach, MD**	Department of Internal Medicine IV, Jena University Hospital, Friedrich-Schiller University Jena, Jena
	**Ansgar W. Lohse, MD**	I. Department of Medicine, University Medical Center Hamburg-Eppendorf, Hamburg
	**Stefano Fusco, MD**	Division of Gastroenterology, Hepatology, Infectious Diseases, Department of Internal Medicine I, University Hospital Tübingen, Tübingen
Germany	**Jost Langhorst, MD**	Department for Internal and Integrative Medicine, Kliniken Essen-Mitte, Essen
	**Jost Langhorst, MD**	Department for Internal and Integrative Medicine, Sozialstiftung Bamberg, Chair for Integrative Medicine, University of Duisburg-Essen, Bamberg
	**Helga Paula Török, MD**	Department of Medicine II, University Hospital, LMU Munich, Munich
Ireland	**Valerie Byrnes, MD**	University College Hospital Galway, Galway

Country	Principal Investigator	Institution
Lithuania	**Juozas Kupcinskas, MD**	Gastroenterology Department and Institute for Digestive Research, Lithuanian University of Health Sciences, Kaunas
Norway		
	**Øistein Hovde, MD, PhD**	Medical Department, Innlandet Hospital Trust, Gjøvik
	**Jørgen Jahnsen, MD**	Akershus University Hospital, Lørenskog
Switzerland	**Luc Biedermann, MD**	Department of Gastroenterology and Hepatology, University Hospital Zürich, Zürich
	**Jonas Zeitz, MD**	Swiss Celiac Center, Center of Gastroenterology, Clinic Hirslanden, Zürich and Department of Gastroenterology and Hepatology, University Hospital Zürich, Zürich
